# High Performances of Artificial Nacre-Like Graphene Oxide-Carrageenan Bio-Nanocomposite Films

**DOI:** 10.3390/ma10050536

**Published:** 2017-05-16

**Authors:** Wenkun Zhu, Tao Chen, Yi Li, Jia Lei, Xin Chen, Weitang Yao, Tao Duan

**Affiliations:** 1 State Key Laboratory Cultivation Base for Nonmetal Composites and Functional Materials, Southwest University of Science and Technology, Mianyang 621010, China; liyi012315@163.com (Y.L.); chenxin1028@126.com (X.C.); tyao@ustc.edu.cn (W.Y.); 2 Engineering Research Center of Biomass Materials, Ministry of Education, Mianyang 621010, China; 18181748525@163.com (T.C.); leijia@gd.swust.edu.cn (J.L.)

**Keywords:** nacre, graphene oxide, carrageenan, self-assembly, nanocomposite films

## Abstract

This study was inspired by the unique multi-scale and multi-level ‘brick-and-mortar’ (B&M) structure of nacre layers. We prepared the B&M, environmentally-friendly graphene oxide-carrageenan (GO-Car) nanocomposite films using the following steps. A natural polyhydroxy polymer, carrageenan, was absorbed on the surface of monolayer GO nanosheets through hydrogen-bond interactions. Following this, a GO-Car hybridized film was produced through a natural drying process. We conducted structural characterization in addition to analyzing mechanical properties and cytotoxicity of the films. Scanning electron microscope (SEM) and X-ray diffraction (XRD) analyses showed that the nanocomposite films had a similar morphology and structure to nacre. Furthermore, the results from Fourier transform infrared spectroscopy (FT-IR), Raman spectroscopy, X-ray photoelectron spectroscopy (XPS) and Thermogravimetric (TG/DTG) were used to explain the GO-Car interaction. Analysis from static mechanical testers showed that GO-Car had enhanced Young’s modulus, maximum tensile strength and breaking elongation compared to pure GO. The GO-Car nanocomposite films, containing 5% wt. of Car, was able to reach a tensile strength of 117 MPa. The biocompatibility was demonstrated using a RAW264.7 cell test, with no significant alteration found in cellular morphology and cytotoxicity. The preparation process for GO-Car films is simple and requires little time, with GO-Car films also having favorable biocompatibility and mechanical properties. These advantages make GO-Car nanocomposite films promising materials in replacing traditional petroleum-based plastics and tissue engineering-oriented support materials.

## 1. Introduction

Evolution over hundreds of millions of years has provided natural biomaterials with perfect structures and functions. Among the numerous natural biomaterials, nacre has received widespread attention, due to its unique layered structure, high strength and outstanding toughness. The structural model of nacre has inspired people to prepare light, highly strong and ultra-tough layered nanocomposites [1,2,3,4]. The main components of natural nacre are aragonite-style calcium carbonate (about 95% wt.) and organic substrates (5% wt.). Nacre has a unique multi-scale, multi-level and orderly ‘brick-and-mortar’ (B&M) composition structure. Specifically, organic substrates firmly hold the calcium carbonate sheets together, playing a role like cement. However, this structure can effectively disperse the pressure imposed on the shell, which is a very favorable mechanical property. The organic substrates of the structured nanocomposite have the effect of dispersing energy, while the layers of inorganic calcium carbonate play a key role in enhancing the mechanical properties of nanostructures.

Over the last few years, inspired by the unique multi-scale and multi-level B&M composition structure in nacre, many studies have reported a series of bio-inspired, highly strong and ultra-tough composites. These composites have been produced using diverse self-assembly techniques, such as layer by layer (LBL) deposition [5,6,7,8,9], vacuum filtration-assisted assembly [10,11,12,13], freeze-drying assembly [14,15,16,17], interface-assisted self-assembly [1,7] and Langmuir–Blodgett assembly [18,19,20]. Two-dimensional inorganic assembly units, such as glass flakes, alumina flakes, graphene oxide, layered double hydroxides, nano-clay and other units, serve as the ‘bricks’, while the high polymers act as the ‘mortar’. It is important to note that graphite oxide (GO) is an ideal two-dimensional inorganic assembly unit for preparation of nacre-like materials. Various assembly techniques have been adopted for the preparation of nacre-like GO-synthetic polymer composite materials. Using the vacuum-assisted self-assembly technique, Putz et al. prepared polymethyl methacrylate(PMMA)-graphene oxide materials, which were found to have a mechanical tensile strength of 180 MPa [21]. Using the self-precipitation assembly technique, Cheng et al. produced 10,12-pentacosadiyn-1-ol(PCDO)-graphene oxide materials with a mechanical tensile strength of 159 MPa [13]. However, few studies focused on the preparation of hybrid nanomaterials using natural high polymers.

Considering the exhaustion of fossil resources in polymer chemical industries and the non-degradable waste from organic polymers, one of the most important trends has been to replace the traditional petroleum-based plastics with natural polymers [8]. Carrageenan (Car), also known as pelvetia silquosa glue, Carrageen glue or Irish moss glue, is a type of polysaccharide extracted from red algae in the sea. Car is a mixture of different types of materials and has special physiological functions, meaning that it has wide applications in the food, medical and chemical industries as well as in biology fields [22]. At present, reports of Car have mainly focused on the edible blend (such as starch, konjac glucan-mannan and chitosan). Furthermore, the water resistance and tensile property of prepared carrageenan food packaging film is poor.

Sijun et al. studied the gel properties of graphene and carrageenan. A schematic diagram has been proposed to explain the effect of ammonia-functionalized graphene oxide (AGO) on the gelation of κ-carrageenan. AGO sheets have been shown to attract a number of κ-carrageenan chains through hydrogen bonding and electrostatic interactions between sulfate groups of κ-carrageenan and amine groups of AGO [23]. Liu et al. conducted experiments examining GO-carrageenan (GO-Car), with the resulting GO-carrageenan (GO-Car) composite being further used as a substrate for biomimetic and cell-mediated mineralization of hydroxyapatite (HA) [22]. However, there is a lack of studies examining the artificial nacre-like graphene oxide-carrageenan bio-nanocomposite films.

Car has numerous active hydroxyls, thus allowing the preparation of GO-Car nanocomposite films as a result of the oxygen-bearing functional groups of GO [24]. In the present study, we used inorganic GO nanosheets to form the ‘bricks’ and carrageenan high-molecular polymers to form the ‘mortar’ for preparation of GO-Car nanocomposite films. Furthermore, we studied the morphology, structure, mechanical properties and cytotoxicity of GO-Car nanocomposite films. GO-Car nanocomposite films may have potential applications in replacing traditional petroleum-based plastics and tissue engineering-oriented support materials.

## 2. Materials and Methods

### 2.1. Chemical Reagents

Graphite powder (99%), potassium persulfate (K_2_S_2_O_8_), phosphorus pentoxide (P_2_O_5_), hydrogen peroxide (H_2_O_2_, 30%), concentrated sulfuric acid (H_2_SO_4_, 96%) and hydrochloric acid (HCl) were provided by Chengdu Kelong Chemical Co., Ltd. (Chengdu, China) and Cheng Du Kelong Chemical Reagent Company (Chengdu, China). We used de-ionized water in this study. Carrageenan was provided by Mianyang Haomao Konjac Food Co., Ltd (Mianyang, China). All the chemicals listed above were analytical reagents and none of them underwent purification treatment before use.

### 2.2. Preparation of GO and Car Solution

GO was prepared using a modified Hummers method as previously described [25,26]. The 1 mg/mL monodisperse and stable GO solution was prepared by putting 0.2 g of graphite oxide powder into 200 mL water, before being dispersed using ultrasonic waves for 60 min. The 1 mg/mL carrageenan solution was obtained by adding 0.1 g of carrageenan powder into 100 mL water, before stirring for 2 h. 

### 2.3. Preparation of GO-Car Nanocomposite Films

The corresponding proportional Car solutions with 0 mL, 2.5 mL, 5 mL and 10 mL were slowly added to the 50 mL GO solution, with this mixed solution being continuously stirred for 1 h. Following this, about 40 to 45 mL GO-Car solutions with different mass ratios were poured into four petri dishes at room temperature to be naturally dried. Finally, 5% GO-Car nanocomposite films, 10% GO-Car nanocomposite films and 20% GO-Car nanocomposite films were obtained.

### 2.4. Characterization

To characterize the GO-Car nanocomposite films, scanning electron microscope (SEM) images were obtained using the Ultra 55 machine (Zeiss, Jena, Germany). The test film was sliced into 10 mm × 100 mm films before SEM scanning. X’Pert PRO (PANalytical, Almelo, The Netherlands) X-ray diffractometer was used to measure the solid X-ray diffraction (XRD) diagram, using Cu K*α* (*λ* = 0.154 nm) radiation with 36 kV and 20 mA as the testing voltage and electric current at 4°/min. The scan range 2*θ* was set at 3°–50° during testing. Nicolet-5700 (Nicolet, New York, NY, USA) was used to scan the Fourier Transform Infrared Spectroscopy (FT-IR) spectrogram in a mode of attenuated total reflectance (ATR), with a wavelength range of 4000–225 cm^−1^. In the temperature range of approximately 40–900 °C, the organic substance and its content in the products was characterized by Thermogravimetric (TG/DTG) at the heating rate of 20 °C/min. X-ray photoelectron spectroscopy (XPS) was measured with the monochromatic light of ALK *α*.h, using a power of 150 W and a beam spot at 500 μm, which was fixed through a 25-eV energy analyzer. For the surface measurement, the core level spectrum was measured from the angle of 90°. The mechanical properties (tensile strength) of composite films were measured using static mechanical testers (Instron 5565A, Beijing, China), with the distance between the two fixtures being 5 mm at a moving speed of 10 mm/min. Films were cut into 23 mm × 5 mm rectangular films before testing.

### 2.5. Cytotoxicity Assays

Mouse peritoneal macrophages, RAW264.7 cells, were grown in Dulbecco’s modified Eagle’s medium (DMEM) supplemented with 10% calf serum (HyClone) and 1% antibiotics (100 Units per mL penicillin and 100 mg/mL streptomycin), before cultured at 37 °C with 5% CO_2_. Disinfected GO-Car nanocomposite films (3 mm × 3 mm) were placed into 24-well plates, with 1 mL DMEM being added into each well. These films were soaked and moistened for 24 h, with the media then being removed from plates. Two milliliters of RAW264.7 cells with a concentration of 2 × 10^6^ per mL and 2 mL of RAW264.7 cell DMEM culture was added to the sample surface in each hole. After this, RAW264.7 cells with film treatment were cultured at 37 °C with 5% CO_2_ for 48 h. The inverted microscope (Nikon ECLIPSE Ti-S, Kanagawa, Japan) was used to observe cell adhesion, cellular morphology and growth situation, with the MTT assay being used to analyze effects of RAW264.7 on proliferation rates and to evaluate cell cytotoxicity of GO-Car composite films following the manufacturer's instructions [9,27].

## 3. Results and Discussion

**Figure 1:**
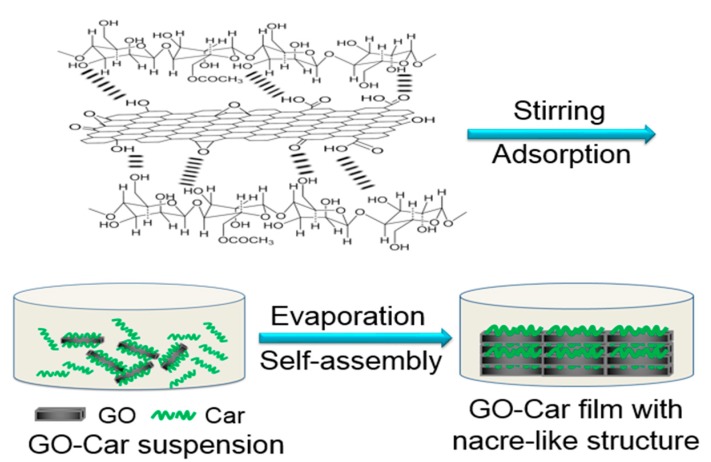
Scheme for fabricating green graphene oxide-carrageenan (GO-Car) composite films.

Figure 1 shows the process designed for preparation of GO-Car composite films. First, we separated monolayer GO nanosheets from the water solution, before adding carrageenan solution into the solution of separated GO nanosheets. This method of adding the solution is intended to facilitate Car molecules absorbing onto the surface of GO nanosheets through hydrogen-bond interactions. Two-dimensional orientations of remarkable cohesive deposition of Car after being naturally dried gave rise to orderly nacre-like structures in GO-Car hybrid films. Compared with the layer-by-layer and other assembly techniques, the whole preparation process was simple and required less time, which facilitates large-scale production. 

**Figure 2:**
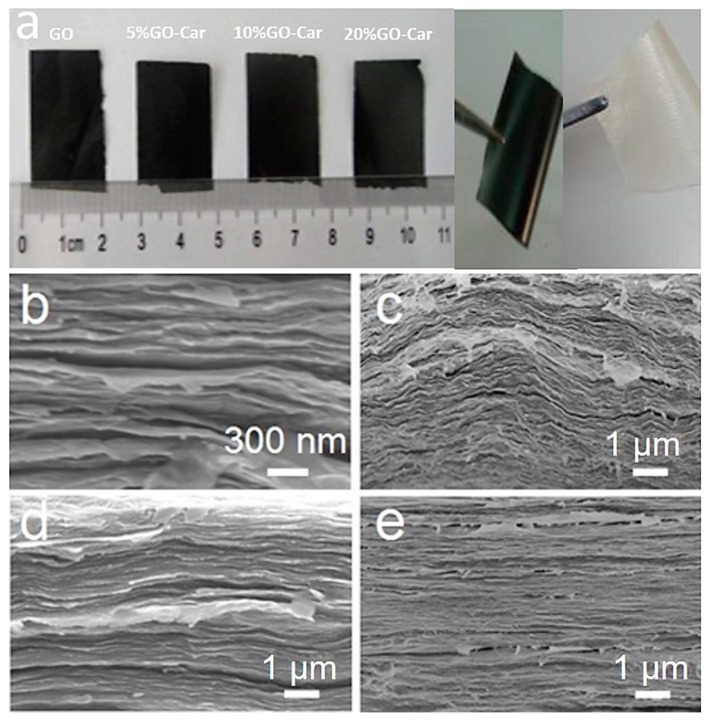
Morphological images of nacre-like GO-Car nanocomposite films: (**a**) Images of GO, Car and GO-Car nanocomposite films. The other parts show the typical SEM images of the fracture surface of GO and GO-Car nanocomposite films at different magnifications: (**b**) Pure GO film; (**c**) 5% GO-Car; (**d**) 10% GO-Car and (**e**) 20% GO-Car.

Nacre-like GO-Car nanocomposite films were prepared through self-evaporation deposition. The images of prepared nanocomposite films are shown in Figure 2a. The slice was soft, with a smooth surface. Figure 2c–e depicts the SEM images of the cross-sections of GO-Car nanocomposite films with different mass ratios. Compared with pure GO films in Figure 2b, there was less space between the GO-Car hybridized nanosheets. The dense parallel stacking and highly oriented layered structure was quite similar to the B&M structure of nacre. The oxygen-bearing functional groups in GO reacted with macromolecular hydroxyls in carrageenan through strong hydrogen-bond interactions. Furthermore, self-evaporation deposition also led to a strong two-dimensional orientation and increased the adhesive action of Car. Both factors contributed to the production of closely layered nacre-like structures, which enhanced the adhesive force among graphene oxide nanosheets.

**Figure 3:**
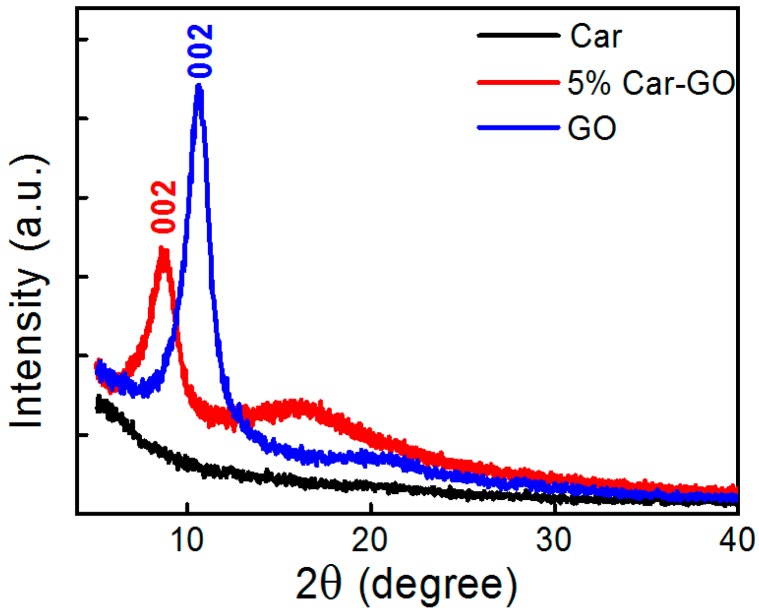
X-ray diffraction (XRD) patterns of GO, Car and 5% GO-Car nanocomposite films.

To investigate the diffraction properties of GO-Car nanocomposite films, we conducted XRD analyses for carrageenan, GO and 5% GO-Car nanocomposite films. As shown in Figure 3, Car mainly existed in an amorphous state, with no obvious characteristic diffraction peaks [28]. Pure graphene oxide films had a diffraction peak at 2*θ* = 10.6°, corresponding to (002) crystal planes of graphite reflection with a 0.84 nm interlayer spacing [29]. For 5% GO-Car nanocomposite films, the characteristic diffraction peak was at 2*θ* = 8.6°, corresponding to (002) crystal planes of graphite reflection with a 1.14 nm interlayer spacing [30]. The above results may be due to the GO nanosheets having absorbed Car macromolecules through hydrogen-bond interactions, resulting in a widened interlayer spacing among graphene oxide nanosheets.

**Figure 4:**
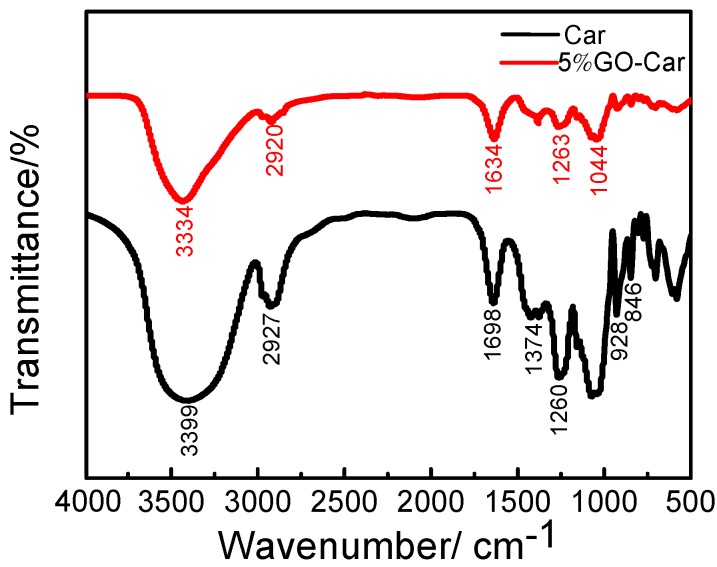
Fourier transform infrared spectroscopy (FT-IR) spectrum of Car and 5% GO-Car nanocomposite film.

According to the results from the Fourier Transform Infrared Spectroscopy (FT-IR) spectrogram (Figure 4) of carrageenan and 5% wt. GO-Car hybrid, there were strong interactions between GO and Car molecules. In the IR spectrum of carrageenan, the absorption peaks at 2927 and 1374 cm^−1^ were the stretching vibration and bending vibration of carbohydrate C–H, respectively. The absorption peaks at 1260, 928 and 846 cm^−1^ were the characteristic absorption peaks of C–O–C and C–O–S stretching vibrations in carrageenan molecules.

After carrageenan was absorbed on GO and formed GO-Car hybrid nanosheets, the C–O–C absorption peaks were at 1044 cm^−1^ and 1263 cm^−1^, while the C=C absorption peak was at 1634 cm^−1^. There was a narrowing in the strong absorption band of OH at 3334 cm^−1^, probably due to the hydrogen bond between the graphene and carrageenan molecules bringing these two molecules closer together. Compared with pure carrageenan, there was no new FT-IR absorption peaks in GO-Car, suggesting that no chemical bond was formed between GO and Car. As the water in the GO-Car solution evaporated, there was an increase in the viscosity of the solution. Carrageenan molecules gradually entangled themselves into a network structure, with the GO nanosheets being encapsulated in the GO layer. Several hydrogen bonds in the original carrageenan molecules were broken, with a new strong interaction being formed between GO and the molecules of carrageenan.

**Figure 5:**
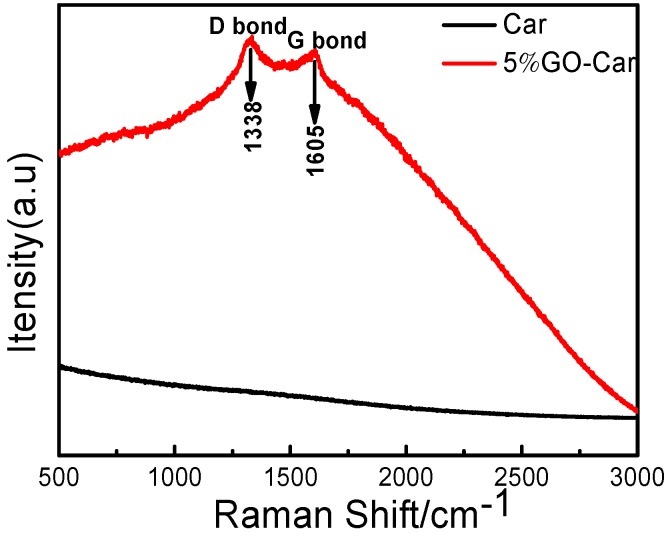
Raman spectrums of Car and 5% GO-Car nanocomposite films.

The Raman spectrums of Car and GO-Car are shown in Figure 5. The G band at 1605 cm^−1^ is characteristic of graphitic carbon layers, corresponding to the tangential vibration of carbon atoms, while the D band at 1338 cm^−1^ indicates a defective graphitic carbon [31]. The intensity ratio of the D band and G band (ID/IG) was 1.02. The D band and G band was clearly observed in the GO-Car nanocomposite films, suggesting the existence of carbon in the composite [32].

**Figure 6:**
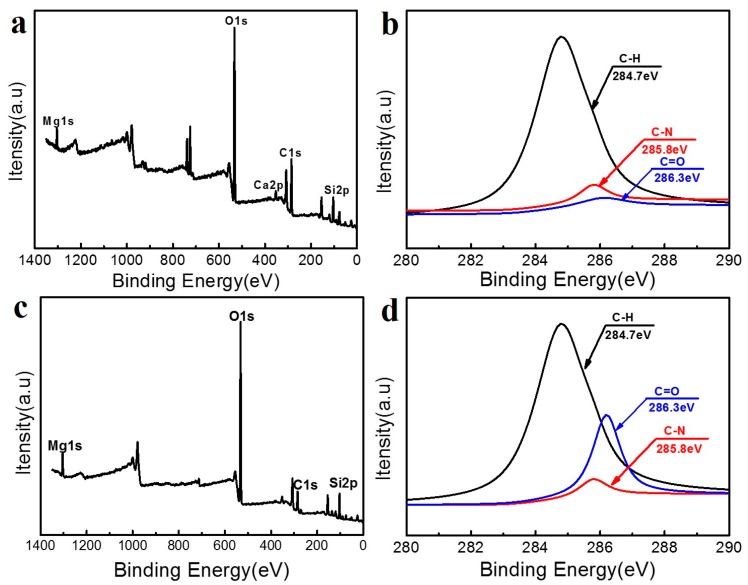
X-ray photoelectron spectroscopy (XPS) spectra of (**a**) Car nanocomposite films; (**b**) Car nanocomposite film with a core of C1s; (**c**) 5% GO-Car nanocomposite films and (**d**) 5% GO-Car nanocomposite film with a core of C1s.

XPS was used in order to further explore the interaction between GO and Car. In the XPS spectrum, the position of each line corresponds to the binding energy of the electrons. The XPS spectra test for Car with a core of C1s is shown in Figure 6b. The C1s peak at 284.7 eV belonged to C–H, while the C1s peak at 285.8 eV belonged to C–N. The peak height of C–H was found to be significantly higher than the peak of C–N, with the C elements in Car being mainly C–H, followed by C–N. The XPS spectra test for GO-Car with a core of C1s is shown in Figure 6d. The C binding energies were 284.7 and 286.3 eV, respectively, which belonged to C–H and C=O. Compared with Figure 6d, the C–N bond is the main component in the C1s of carrageenan (Figure 6b), because the carrageenan is a galactose formed by two galactoses. In comparison, C=O occupies the main position in GO-Car (Figure 6d), which may be the result of graphene oxide having played a certain role in promotion. There was no obvious change in the intensity of each peak, suggesting that there was no new chemical bond formation during physical mixing. It was thought that physical mixing led to the formation of new hydrogen bonds.

**Figure 7:**
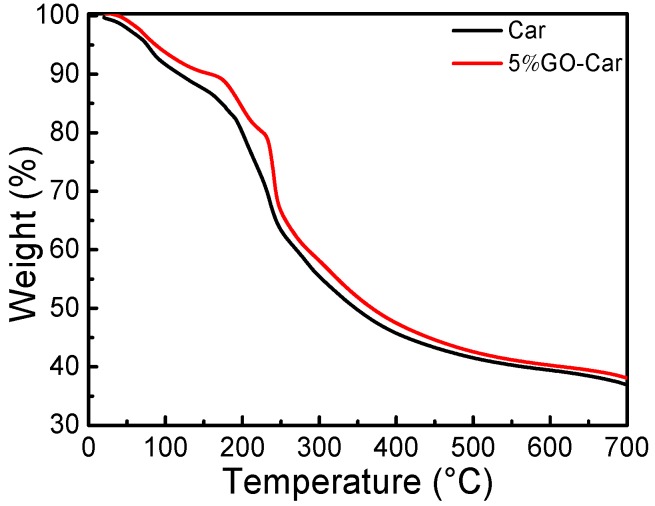
Thermogravimetric (TG/DTG) spectra of Car and 5% GO-Car nanocomposite film.

It can be seen from Figure 7 that the thermal decomposition of Car film began at 65 °C, while the thermal decomposition of the GO-Car nanocomposite films began at 72 °C. The thermal stability of GO-Car nanocomposite films was better than the pure Car film. At 100 °C, there was no significant degradation of weight loss, which might be due to the loss of water molecules in the matrix. It can be seen from Figure 7 that the thermal decomposition occurred in the range of 100–350 °C. There was a loss of most of the oxygen-containing functional groups, hastening the weight decline. In GO-Car nanocomposite films, the oxygen-containing functional groups decomposed into CO and CO_2_ [31]. When the temperature exceeded 200 °C, the polymer chain of carrageenan was destroyed. After 500 °C, the thermal weight loss was very slow, with the thermal decomposition gradually becoming stabilized.

**Figure 8:**
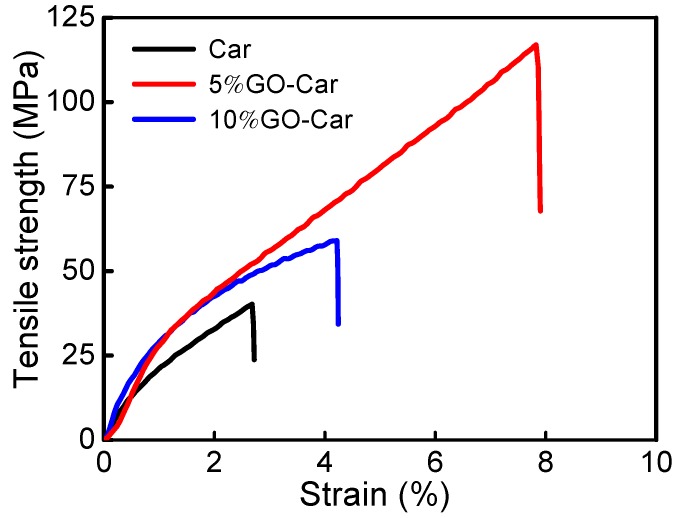
Typical stress-strain curves of Car and GO-Car nanocomposite films.

In order to demonstrate the enhanced mechanical properties resulted from the nacre-like micro structure, we characterized the tensile strengths of GO-Car nanocomposite films with different mass ratios, using pure graphene oxide as the control group (Figure 8). For the 5% GO-Car nanocomposite films, the maximum tensile strength was 117 MPa and the breaking elongation was 7.82%, meaning that it was 190.90% and 191.79% higher than the corresponding values of pure GO. For the 10% GO-Car nanocomposite films, the maximum Young’s modulus was 21.87 GPa, which was 24.26% higher than that of Car. These significant improvements can be attributed to the strong hydrogen-bond interactions between GO nanosheets and Car macromolecules in addition to the layered structure, which was similar to natural nacre.

**Figure 9:**
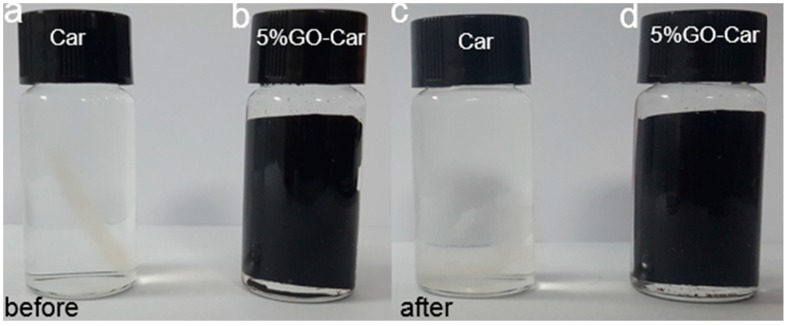
Photographs of different films being immersed in distilled water for different amounts of time: (**a**) Car film for 5 min; (**b**) 5% GO-Car nanocomposite film for 5 min; (**c**) Car film for 7 days and (**d**) 5% GO-Car nanocomposite film for 7 days.

Compared with the pure Car films, there was a dramatic increase in the stability of the hybrid film in the wet environment. As shown in Figure 9, the pure Car nanocomposite films apparently degraded after being immersed in water for seven days. The film was broken into many small pieces. However, for the GO-Car nanocomposite films, there was negligible degradation and the film was still maintained with initial robustness.

**Figure 10:**
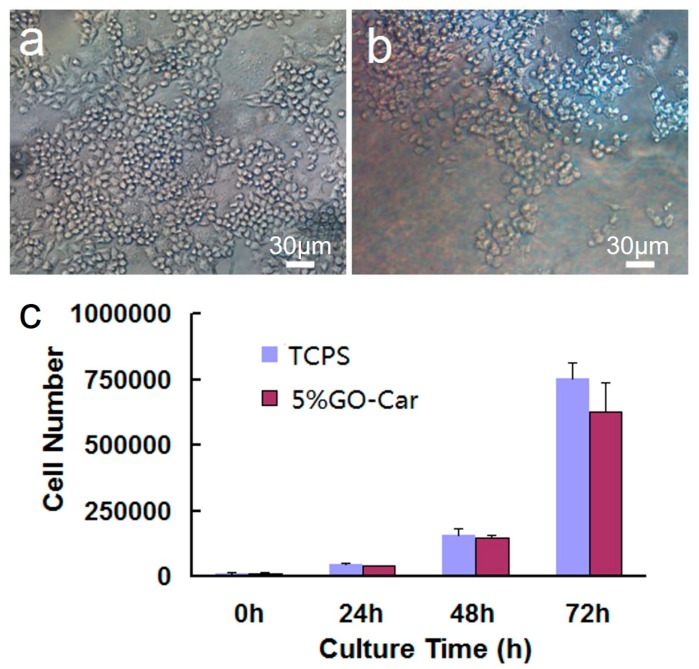
(**a**,**b**) Phase contrast and fluorescence images of human umbilical vein endothelial cells (HUVECs) grown on tissue culture polystyrene (TCPS) plates and a GO-Car nanocomposite films coated surface for 48 h; (**c**) RAW264.7 Cell viability measured by MTT assay after being cultured for 0, 24, 48 and 72 h.

The cytotoxicity of GO-Car nanocomposites was evaluated through growing RAW264.7 cell on the films for 48 h. Figure 10 depicts the influences of GO-Car nanocomposite films on cellular morphology and cell proliferation, using the inverted microscope (Nikon ECLIPSE Ti-S, Kanagawa, Japan) and MTT colorimetric assay. Cells adhered on the surface of nanocomposite films had clear outlines, with the oval shape being similar to the control group tissue culture polystyrene (TCPS). These results indicated that GO-Car nanocomposite films did not affect cellular morphology and cell proliferation. Therefore, the nanocomposite films with outstanding cell biocompatibility might have a promising application in the field of tissue engineering-oriented support materials.

## 4. Conclusions

Inspired by nacre, our study used inorganic GO nanosheets as the ‘bricks’ and carrageenan (natural polymeric compound) as the ‘mortar’ to mimic the layered structure of nacre for preparing the GO-Car nanocomposite films. The results from SEM, FT-IR, Raman, XRD, TG/DTG, XPS, Raman and the Instron tensile tester indicated that carrageenan molecules can closely bond with GO nanosheets through hydrogen-bond interactions and form nacre-like nanocomposite films. Young’s modulus, maximum tensile strength and breaking elongation were significantly improved in GO-CAR nanosheets when compared with pure GO films. Furthermore, such films have favorable biocompatibility, indicating a potential application in replacing the traditional petroleum-based plastics and tissue engineering-oriented support materials.
